# A Longitudinal Quasi-Experimental Evaluation of a Cognitive-Behavioral Intervention in Teachers: Changes in Psychosocial Risks, Burnout, Depression, and Salivary Cortisol

**DOI:** 10.3390/bs16091668

**Published:** 2026-09-16

**Authors:** Ester Grau-Alberola, Pedro R. Gil-Monte, Bruno Ribeiro do Couto, Hugo Figueiredo-Ferraz

**Affiliations:** 1 Facultad de Psicología, Universidad Católica de Valencia “San Vicente Mártir”, 46100 Valencia, Spain; ester.grau@ucv.es; 2 Unidad de Investigación Psicosocial de la Conducta Organizacional (UNIPSICO), Universitat de València, 46010 Valencia, Spain; pedro.gil-monte@uv.es; 3 Institute of Biomedical Research of Murcia, Virgen de la Arrixaca University Hospital, 30120 Murcia, Spain; bruno.ribeiro@um.es; 4 Department of Human Anatomy and Psychobiology, Faculty of Psychology, University of Murcia, 30100 Murcia, Spain; 5 Facultad de Ciencias Sociales y Jurídicas, Universidad Internacional de Valencia (VIU), 46002 Valencia, Spain

**Keywords:** psychosocial risks, burnout, health problems, depressive symptoms, cortisol, cognitive–behavioral intervention

## Abstract

Burnout is recognized in the ICD-11 as an occupational phenomenon resulting from chronic work-related stress. In educational settings, psychosocial risks—particularly high job demands and insufficient resources—have been associated with burnout, depressive symptoms, and physiological stress dysregulation. This longitudinal quasi-experimental study examined changes associated with participation in a multicomponent cognitive–behavioral intervention among teachers. The sample comprised 66 primary and secondary school teachers (30 training, 36 non-training), further stratified according to high or low baseline levels of stress-related health problems. Measures included resource availability, self-efficacy, interpersonal conflicts, work overload, burnout, depressive symptoms, and salivary cortisol, assessed as total morning cortisol output using the area under the curve with respect to ground (AUCg), at baseline (T1), post-intervention (T2), and four-month follow-up (T3). The clearest pattern of statistically significant within-group changes was observed among trained teachers with high baseline stress-related health problems (TG-HHP; *n* = 13). From T1 to T2, this subgroup showed increased resource availability and self-efficacy and reductions in burnout (1.63 to 1.33; *p* = 0.013, d = 0.81), depressive symptoms (46.92 to 42.40; *p* = 0.032, d = 0.67), and morning cortisol AUCg (410.01 to 306.50; *p* = 0.018, d = 0.78). At T3, statistically significant changes relative to baseline were observed across all study variables. These findings provide preliminary evidence that participation in a structured cognitive–behavioral intervention may be associated with psychosocial, psychological, and physiological improvements, particularly among teachers with elevated baseline stress-related health problems.

## 1. Introduction

The OSH Pulse 2025 survey, conducted by the European Agency for Safety and Health at Work (EU-OSHA), provides a concerning picture of occupational health across Europe. Among European workers, stress, anxiety, and depression constitute the second most common category of work-related health problems. Furthermore, 45% of employees reported having been consulted about stressful aspects of their work, while 40% reported having access to counseling or psychological support. The survey also identifies workers in the education sector as one of the occupational groups most affected by work-related stress and psychosocial hazards (Belli & De Keulenaer, 2025).

Teaching ranks among the occupations most exposed to psychological strain, largely because of its combination of emotional demands, social responsibility, and structural pressures within the educational system (Agyapong et al., 2022). What often begins as a normal adaptive response to classroom challenges can evolve into a chronic state of stress sustained by unfavorable psychosocial working conditions (Kyriacou, 2001).

According to international organizations, psychosocial risks refer to aspects of work design, organization, and interpersonal relations that have the potential to cause social, psychological, or physical harm (European Agency for Safety and Health at Work, 2023). In line with the Job Demands–Resources (JD–R) model (Demerouti et al., 2001), teacher stress and exhaustion arise from an imbalance between high job demands and insufficient personal or organizational resources. Within the educational context, several psychosocial risks contribute to this imbalance, including work overload (Figueiredo-Ferraz et al., 2021), interpersonal conflicts (Vogel & Schwabe, 2016), lack of resources, and low perceived self-efficacy (Bakker & Demerouti, 2017). Prolonged exposure to such psychosocial pressures gradually transforms an initially adaptive stress response into a maladaptive and pathological process, undermining emotional regulation and physiological stability and paving the way for burnout and depressive reactions (Ghasemi et al., 2024; Maslach & Leiter, 2016). In this sense, chronic stress among teachers is not an isolated phenomenon but rather the early expression of a broader deterioration process that culminates in burnout (Figueiredo-Ferraz et al., 2021).

In this context, burnout syndrome has gained increasing relevance as a critical occupational health problem. Burnout is currently recognized in the ICD-11 (World Health Organization, 2022) as an occupational phenomenon and health problem, rather than as a medical condition; it results from chronic workplace stress that has not been successfully managed (Maslach & Leiter, 2016; World Health Organization, 2022). It occurs particularly among professionals who provide services through sustained direct contact with clients, patients, students, or other service recipients (Gil-Monte, 2005), with important consequences for both individual well-being and organizational functioning (Madigan et al., 2023). According to Gil-Monte (2005), burnout is a non-psychiatric syndrome characterized by cognitive impairment (i.e., loss of enthusiasm for work), emotional deterioration (i.e., psychological exhaustion), and attitudes and behaviors characterized by indifference, emotional distancing, and negative treatment of service recipients (i.e., indolence). In addition, in some cases, negative attitudes at work—especially toward the people with whom workers establish professional relationships—may lead to intense feelings of guilt.

Research indicates that burnout may manifest in distinct profiles (Guidetti et al., 2018; Leiter & Maslach, 2016; Yaghmour et al., 2024). Gil-Monte (2005) identifies two such profiles. In Profile 1, individuals experiencing burnout may still adapt to their work environment, as the syndrome does not prevent them from performing their work tasks, although the quality of the service provided may be suboptimal for clients and the organization. Behaviors such as indolence, cynicism, or depersonalization serve as coping mechanisms that mitigate the effects of the syndrome. In Profile 2, the onset of guilt may initiate a vicious cycle that exacerbates burnout symptoms, leading to increased severity. Over the medium to long term, this process may result in health deterioration, increased absenteeism, and greater substance use (Gil-Monte, 2012; Misiolek-Marín et al., 2020; Olivares-Faúndez et al., 2014; Rabasa et al., 2016). This phenomenon is particularly pronounced among education professionals, a group consistently identified as highly susceptible to the cumulative effects of emotional demands, moral values, and organizational pressures (Brandão et al., 2025; Figueiredo-Ferraz et al., 2016; Skaalvik & Skaalvik, 2021; Tuxford & Bradley, 2015). The physiological and psychological manifestations of burnout among teachers include cortisol dysregulation (Madigan et al., 2023), cognitive failures affecting performance and classroom functioning (Linden et al., 2005), poorer perceived health, and depressive symptoms (Martínez et al., 2020; Salvagioni et al., 2017).

The distinction between burnout and depression is partially supported by empirical research (Tavella et al., 2023). Although they are positively correlated, evidence consistently demonstrates that they are distinct constructs (Leiter & Durup, 1994) and clearly differentiated phenomena (Toker & Biron, 2012), with burnout representing an independent diagnostic entity rather than a form of depression (Koutsimani et al., 2019; Parker & Tavella, 2021; Tement et al., 2016). Burnout may precede work-related depression, co-occur with anxiety, or subsequently be accompanied by depressive symptoms (Baka et al., 2025; Figueiredo-Ferraz et al., 2021); however, this does not imply conceptual equivalence. Notably, the ICD-11 classifies burnout as an occupational phenomenon distinct from medical or psychiatric disorders, reinforcing its unique status. Depression represents a significant mental health outcome associated with chronic occupational stress in the teaching profession, with prevalence rates ranging from 4% to 77% (Agyapong et al., 2022).

The demanding nature of educational work—characterized by emotional involvement, classroom management challenges, administrative workload, and continuous interpersonal interactions—may increase teachers’ vulnerability to depressive symptomatology (Pikó et al., 2025; Steinhardt et al., 2011). Empirical research conducted across various educational contexts indicates that a substantial proportion of teachers report moderate to high levels of depressive symptoms (Ma et al., 2022; Nakada et al., 2016; Silva et al., 2021), often in association with persistent exposure to work-related stressors and adverse psychosocial working conditions (Ruiz-Ruano García et al., 2025; Schonfeld et al., 2017). These conditions may gradually undermine emotional regulation, motivation, and cognitive functioning, contributing to the development of depressive states over time (Lei et al., 2025; Steinhardt et al., 2011).

Depression among teachers has been associated with a wide range of negative consequences, including reduced work engagement, lower job satisfaction, impaired cognitive performance, and diminished instructional effectiveness (Schonfeld et al., 2017; Xiao & Zheng, 2025). Beyond its impact on teachers’ personal well-being, depressive symptomatology may also negatively affect classroom dynamics and the overall quality of the educational environment (Schonfeld et al., 2017). Given the strong relationship between chronic occupational stress and depressive outcomes (Agyapong et al., 2022; Steinhardt et al., 2011), increasing attention has been directed toward understanding the physiological mechanisms underlying this process, particularly the role of the hypothalamic–pituitary–adrenal (HPA) axis and cortisol regulation (de Mello et al., 2003; Joseph & Golden, 2017; Lei et al., 2025).

Chronic exposure to psychosocial stressors activates the hypothalamic–pituitary–adrenal (HPA) axis, a principal neuroendocrine system involved in the physiological regulation of stress (Lei et al., 2025; Varghese & Brown, 2001). When individuals perceive environmental demands as threatening or difficult to control, the hypothalamus stimulates the release of corticotropin-releasing hormone (CRH), which triggers the secretion of adrenocorticotropic hormone (ACTH) by the pituitary gland (Amsterdam et al., 1989; Thomson & Craighead, 2008). This process ultimately leads to the release of cortisol from the adrenal cortex. Under normal conditions, cortisol plays an adaptive role by mobilizing energy resources and facilitating the organism’s response to acute stress (Kudielka et al., 2006). However, when stressors become chronic, as frequently occurs in environments characterized by persistent psychosocial demands, sustained activation of the HPA axis may lead to dysregulation of cortisol secretion (Chandola et al., 2010; Wolfram et al., 2013).

A growing body of research suggests that prolonged exposure to adverse psychosocial working conditions, including high workload, interpersonal conflicts, and low control over work demands, is associated with alterations in cortisol regulation (Karlson et al., 2011; Maina et al., 2008). Such dysregulation has been linked to chronic stress-related conditions, including burnout and depression (Danhof-Pont et al., 2011; Rothe et al., 2020). Evidence indicates that individuals experiencing high levels of burnout may exhibit abnormal cortisol patterns, reflecting impaired stress regulation and physiological exhaustion (Kudielka et al., 2006; Ungurianu & Marina, 2025; Verhaeghe et al., 2012). Similarly, dysregulated cortisol secretion has been consistently associated with depressive symptomatology (Kunugi et al., 2015; Thomson & Craighead, 2008; Varghese & Brown, 2001), suggesting that alterations in HPA-axis functioning may constitute a biological pathway linking chronic psychosocial stress to both burnout and depression (Rothe et al., 2020). Psychological interventions grounded in cognitive–behavioral approaches have been widely applied to reduce occupational stress, burnout, and depressive symptoms in helping professions (Agyapong et al., 2023; Paudel et al., 2022).

Traditional cognitive–behavioral therapy (CBT) focuses on identifying and modifying maladaptive cognitive patterns, improving emotional regulation, and strengthening coping strategies when individuals face chronic stressors (Ghasemi et al., 2023; Żołnierczyk-Zreda, 2005). Within the educational context, several intervention programs integrating these approaches have shown promising results in improving teachers’ psychological well-being and reducing symptoms of stress, burnout, and depression (Bonde et al., 2022; de Carvalho et al., 2021; Lensen et al., 2024; Matos et al., 2022). In line with these approaches, the present intervention program combined cognitive, emotional, physiological, and psychosocial techniques specifically adapted to the teaching profession.

The program included psychoeducation on work stress and psychosocial risks among teachers, relaxation techniques aimed at physiological stress regulation, emotional management strategies, and cognitive techniques such as internal dialogue management and cognitive restructuring (Ghasemi, 2023; Oishi et al., 2018). In addition, participants received training in problem-solving skills, personal and professional self-organization, and strategies designed to strengthen social support and role awareness within the educational context (Schnaider-Levi et al., 2020; Żołnierczyk-Zreda, 2005). By addressing both cognitive and behavioral coping processes, this type of structured intervention seeks to reduce the psychological impact of occupational stress while promoting adaptive resources that protect teachers’ mental health (Agyapong et al., 2023; Bian & Jiang, 2025; Dvořáková et al., 2024; Jennings & DeMauro, 2017; Matos et al., 2024).

Teachers’ psychosocial health is not exclusively an occupational issue; it is also closely connected to the educational process. Sustained teacher distress may undermine the quality of classroom interactions, teachers’ emotional availability to students, the management of interpersonal and classroom dynamics, and, ultimately, students’ well-being and learning experiences (Madigan et al., 2023; Schonfeld et al., 2017; Skaalvik & Skaalvik, 2021). From a behavioral and occupational-health perspective, identifying which psychosocial and health-related outcomes are responsive to intervention, and among which teachers these changes are most evident, is relevant to the design of workplace prevention and professional development programs adapted to differences in teachers’ initial vulnerability.

Previous interventions targeting teacher stress and burnout have frequently relied primarily on self-reported outcomes and have often been evaluated without examining whether their effects differ according to participants’ baseline levels of stress-related health problems (Denuwara et al., 2022; Paudel et al., 2022). Evidence integrating psychosocial resources and demands, mental health indicators, physiological measures, and longitudinal follow-up remains comparatively limited. Examining baseline vulnerability may therefore provide a more differentiated understanding of who is most likely to benefit from an intensive intervention and may inform more targeted approaches to teacher well-being.

Accordingly, the present longitudinal quasi-experimental study examined changes associated with participation in a structured multicomponent cognitive–behavioral intervention among teachers. Specifically, it assessed perceived job resources and demands, burnout, depressive symptoms, and salivary cortisol at baseline, post-intervention, and follow-up, distinguishing between teachers with high and low baseline levels of stress-related health problems. In doing so, the study sought to provide preliminary evidence relevant to the development of needs-based teacher well-being and stress-prevention programs in educational settings.

The intervention was designed to strengthen personal and occupational resources while modifying the appraisal and management of job demands. Accordingly, increases in perceived resource availability and self-efficacy, together with reductions in workload and interpersonal conflicts, were expected among trained teachers with greater baseline vulnerability. Because chronic occupational stress is associated with burnout, depressive symptoms, and dysregulation of the hypothalamic–pituitary–adrenal axis, improvements in these psychological and physiological outcomes were also expected in this subgroup. By contrast, teachers with lower baseline levels of stress-related health problems were expected to show less change because of their more favorable initial status and, consequently, their more limited scope for improvement. No systematic change was expected among participants who did not receive the intervention. Therefore, the following hypotheses were tested:

**Hypothesis** **H1.** *Trained teachers with high baseline levels of stress-related health problems were expected to show increases in perceived resource availability and self-efficacy across the assessment period.*

**Hypothesis** **H2.** *Trained teachers with high baseline levels of stress-related health problems were expected to show reductions in perceived workload and interpersonal conflicts across the assessment period.*

**Hypothesis** **H3.** *Trained teachers with high baseline levels of stress-related health problems were expected to show reductions in burnout and depressive symptoms across the assessment period.*

**Hypothesis** **H4.** *Trained teachers with high baseline levels of stress-related health problems were expected to show reductions in salivary cortisol levels across the assessment period.*

**Hypothesis** **H5.** *A less consistent pattern of change was expected among trained teachers with low baseline levels of stress-related health problems and among teachers who did not participate in the intervention.*

## 2. Materials and Methods

### 2.1. Participants

The study followed a quasi-experimental longitudinal design involving teachers working in primary and secondary educational centers. The total sample consisted of 66 teachers employed in primary and secondary schools. Of the participants, 58 were women (87.9%) and 8 were men (12.1%). The mean age was 46.42 years (SD = 9.32), with ages ranging from 29 to 64 years. Participants reported an average of 20 years of professional experience (SD = 8.67), ranging from 3 to 37.67 years.

Participants were recruited from educational institutions that voluntarily agreed to collaborate in the research project. Because participation in the intervention program was voluntary, the study adopted a quasi-experimental design rather than a randomized experimental design. The sample comprised two groups: a training group (TG) of 30 teachers who participated in the intervention program and a non-training group (NTG) of 36 teachers who did not receive the intervention.

Group comparability was examined using chi-square tests of independence for the following control variables, including sex, age group, years of professional experience (grouped), workplace location (urban vs. metropolitan area), regular physical exercise, daily alcohol consumption, smoking habits, medication intake (e.g., statins, antidepressants, anxiolytics, anti-inflammatory drugs, antirheumatic drugs, calcium derivatives, iron supplements, vitamins, and hormonal treatments), levels of burnout, and psychosomatic disorders associated with work stress.

For the planned subgroup analyses, participants were stratified according to their baseline scores on the Psychosomatic Disorders Scale at T1. The sample median (Mdn = 1.50) was used as the cut-off point. This procedure resulted in four analytical subgroups: (1) the trained group with high stress-related health problems (TG-HHP; *n* = 13); (2) the trained group with low stress-related health problems (TG-LHP; *n* = 17); (3) the non-trained group with high stress-related health problems (NTG-HHP; *n* = 20, *n* = 19 at follow-up); and (4) the non-trained group with low stress-related health problems (NTG-LHP; *n* = 16, *n* = 15 at follow-up). The differences between baseline and follow-up subgroup sizes correspond to participants who did not complete the follow-up assessment, because they did not provide the questionnaire or a valid salivary cortisol sample at that wave. These participants were excluded from the corresponding analyses.

### 2.2. Intervention Program

#### 2.2.1. Program Rationale, Structure, and Implementation

Given that burnout symptoms involve cognitive deterioration (low enthusiasm toward work), emotional or affective deterioration (psychological exhaustion and, in some individuals, high levels of guilt), and attitudinal or behavioral deterioration (indolence) (Gil-Monte, 2005), the intervention program included psychological and psychosocial techniques aimed at addressing deterioration in these domains.

It was also necessary to include techniques for managing psychosomatic problems arising from work-related stress (Gil-Monte, 2014) and burnout (Gil-Monte, 2005), given the frequency with which these problems occur (Ahola et al., 2017). Therefore, two relaxation-training sessions were included to address psychosomatic problems associated with anxiety. This perspective, which recommends addressing burnout through programs that incorporate a variety of techniques, has been supported in the literature (Wiederhold et al., 2018).

To select the techniques included in the intervention program, a review of the literature was conducted. This review identified relaxation techniques, cognitive–behavioral techniques (Ahola et al., 2017; Maricuţoiu et al., 2016), and social support interventions (Awa et al., 2010) as being among the most effective approaches. In addition, discussion sessions were held with non-academic psychology professionals experienced in the treatment of burnout to identify the intervention methods most frequently applied in professional practice.

The training program lasted three months and consisted of 10 weekly sessions, each lasting 2.5 h. The intervention was delivered face-to-face at the university by two clinical psychologists, with the support of three organizational psychologists. The same professional team was involved throughout the 10-session program.

To facilitate implementation, the 30 participants in the training condition were divided into two groups of 15. Each weekly session was therefore delivered twice, on separate days, using the same content, materials, and procedures for both groups. The intervention followed a standardized protocol specifying the objectives, content, and activities of each session. Program implementation was supervised to ensure adherence to the established protocol and consistency across the two training groups. All 10 sessions were delivered as planned.

#### 2.2.2. Intervention Content

The program had six objectives: (1) to modify the cognitive schemas associated with participants’ negative professional self-evaluation; (2) to provide participants with strategies for developing emotional regulation skills; (3) to improve strategies for coping with work-related stress and its consequences, particularly those related to burnout syndrome; (4) to develop social skills and adequate levels of self-efficacy to properly manage interpersonal relationships at work, as well as expectations and behaviors related to the work role; (5) to develop the ability to negotiate the work role; and (6) to promote social support in the workplace.

The intervention consisted of a cognitive–behavioral training program designed to reduce occupational stress, burnout, and depressive symptoms among teachers. It included the following components (Gil-Monte, 2019b):

(a) Psychoeducation about psychosocial risks and occupational stress in teaching, including the consequences of stress for health, burnout, and cortisol regulation (Gil-Monte, 2014);
(b) Relaxation-training session I, focused on techniques for reducing physiological activation and managing psychosomatic symptoms associated with anxiety through Ericksonian hypnosis (O’Hanlon, 1993);
(c) Emotional management, including strategies derived from emotion-focused therapy to regulate emotional responses and manage feelings such as guilt (Greenberg & Paivio, 2000);
(d) Internal dialogue management, involving the identification and modification of maladaptive self-talk associated with stressful work situations, using principles of transactional analysis (Berne, 2011; Román, 1994);
(e) Problem-solving training, based on rational problem-solving models aimed at improving coping strategies when facing work-related stressors (LET Program; Gordon, 1980);
(f) Personal and professional self-organization, focusing on goal setting, time management, and the organization of work activities (Briese-Neumann, 1998);
(g) Cognitive restructuring, including the identification and modification of irrational beliefs according to principles of rational-emotive behavior therapy and cognitive therapy (Beck, 2000; Ellis & Grieger, 1990; Wright et al., 2006);
(h) Work-role negotiation, aimed at clarifying job expectations and negotiating responsibilities within the organizational context (Harrison, 1972);
(i) Relaxation-training session II, focused on managing psychosomatic symptoms related to work stress through Ericksonian hypnosis (O’Hanlon, 1993); and;
(j) Social support at work, including training in strategies for offering and receiving support within the workplace (Peterson et al., 2008).

The intervention integrated cognitive–behavioral techniques, emotional regulation strategies, relaxation training, and organizational coping skills, aiming to modify both the cognitive appraisal of job stressors and the availability of psychosocial resources in the workplace.

### 2.3. Instruments

Instruments from the UNIPSICO battery were used to assess psychosocial risk factors and their consequences in the workplace (Gil-Monte, 2016a, 2016b).

#### 2.3.1. Sociodemographic Variables

The first section of the questionnaire collected sociodemographic, occupational, and personal information. Participants generated an anonymous six-digit identification code based on their surname initials and year of birth. This code allowed responses to be matched across the three measurement waves while preserving anonymity.

#### 2.3.2. Psychosomatic Disorders

To stratify participants according to baseline vulnerability, psychosomatic disorders were assessed using the nine-item Psychosomatic Disorders Scale from the UNIPSICO battery (Gil-Monte, 2016a; α = 0.88 at T1). The scale assesses the frequency of work-related psychosomatic symptoms, including headaches, musculoskeletal pain, sleep problems, anxiety, and illness-related complaints.

Participants responded using a five-point frequency scale ranging from 0 (never) to 4 (very frequently: every day). An example item is “Do you have headaches?” The sample median at T1 (Mdn = 1.50) was used as the cut-off point to classify participants as having high or low baseline levels of psychosomatic disorders.

This stratification was theoretically motivated by the study’s focus on examining patterns of change among teachers with different levels of baseline vulnerability. The sample median was used as a pragmatic, sample-based criterion to distinguish relatively higher and lower baseline levels of stress-related psychosomatic symptoms. This categorization necessarily reduces information from the continuous measure, a limitation that was considered when interpreting the findings.

#### 2.3.3. Job Resources

(a) Availability of resources. Availability of resources was assessed using the seven-item Availability of Resources Scale from the UNIPSICO battery (Gil-Monte, 2016b; α = 0.77 at T1, α = 0.81 at T2, and α = 0.81 at T3). The scale evaluates the extent to which workers perceive that they have adequate organizational resources to perform their tasks, including authority or influence at work, personnel and material resources, technological resources, appropriate protective measures, and access to other organizational areas.

(b) Self-efficacy. Self-efficacy was assessed using the 10-item Self-Efficacy Scale from the UNIPSICO battery (Gil-Monte & García-Juesas, 2008; α = 0.90 at T1, α = 0.92 at T2, and α = 0.93 at T3). The scale evaluates workers’ beliefs regarding their ability to cope effectively with job demands. An example item is “Because of my abilities and resources, I can successfully cope with unexpected situations.”

#### 2.3.4. Job Demands

(a) Workload. Workload was assessed using the six-item Workload Scale from the UNIPSICO battery (Gil-Monte, 2016a; α = 0.76 at T1, α = 0.80 at T2, and α = 0.81 at T3). The scale assesses both quantitative and qualitative workload. Quantitative workload refers to the number of tasks that must be completed within a given period, whereas qualitative workload refers to the perceived difficulty of those tasks relative to the time and resources available. Example items are “Do you find that you do not have enough time to complete your work?” and “Do you feel that the work you are required to perform is too difficult for you?”

(b) Interpersonal conflicts. Interpersonal conflicts were assessed using the six-item Interpersonal Conflicts Scale from the UNIPSICO battery (Gil-Monte, 2016a; α = 0.78 at T1, α = 0.77 at T2, and α = 0.78 at T3). The scale measures the frequency of conflicts experienced with supervisors, colleagues, other employees within the organization, and students. An example item is “How often do you experience conflicts with your colleagues?”

Participants rated the items on all these scales using a five-point frequency scale ranging from 0 (never) to 4 (very frequently: every day).

#### 2.3.5. Stress-Related Health Outcomes

(a) Burnout. Burnout was assessed using the Spanish Burnout Inventory (SBI; Gil-Monte, 2019a). The instrument consists of 20 items distributed across four dimensions representing the core components of the burnout process among service professionals.

The four dimensions assessed were: (1) Enthusiasm toward the Job, comprising five items (α = 0.87 at T1, α = 0.90 at T2, and α = 0.87 at T3); (2) Psychological Exhaustion, comprising four items (α = 0.89 at T1, α = 0.90 at T2, and α = 0.81 at T3); (3) Indolence, comprising six items (α = 0.69 at T1, α = 0.82 at T2, and α = 0.68 at T3); and (4) Guilt, comprising five items (α = 0.85 at T1, α = 0.85 at T2, and α = 0.79 at T3).

Enthusiasm toward the Job reflects the individual’s desire to achieve work-related goals because work represents a source of personal satisfaction and professional fulfillment. Psychological Exhaustion refers to emotional and physical fatigue resulting from continuous interaction with people who generate or present problems in the work context. Indolence represents the development of cynical attitudes, indifference, or emotional distancing toward the recipients of the services provided by the organization. Guilt captures the emergence of feelings of guilt associated with negative attitudes or behaviors developed at work, particularly toward individuals with whom professional relationships have been established.

The SBI is widely used in occupational health research and allows the identification of burnout profiles characterized by the progressive deterioration of cognitive, emotional, and attitudinal functioning at work. Participants rated the items using a five-point frequency scale ranging from 0 (never) to 4 (very frequently: every day).

A total burnout (SBI) score was computed by summing the scores of the Psychological Exhaustion, Indolence, and Enthusiasm toward the Job subscales—after reverse-scoring the Enthusiasm toward the Job subscale—and dividing by the total number of items (15). The Guilt subscale was not included in the total score. Higher scores therefore indicate higher levels of burnout, and this composite was the burnout variable used in all analyses (Gil-Monte, 2019a).

(b) Depressive symptoms. Depressive symptoms were assessed using the Spanish adaptation of the 20-item Zung Self-Rating Depression Scale (SDS; Zung, 1965; Gil-Monte, 2012). Participants rated the frequency of each symptom using a four-point scale ranging from 1 (rarely or never) to 4 (almost always or always). An example item is “I feel downhearted and blue.” Internal consistency was α = 0.85 at T1, α = 0.85 at T2, and α = 0.85 at T3.

(c) Salivary cortisol levels. Morning salivary cortisol was sampled at each measurement wave by collecting three samples on a working day using Salivette devices: immediately upon awakening, 15 min later, and 30 min after awakening (Chida & Steptoe, 2009; Dedovic & Ngiam, 2015; Fries et al., 2009). Samples were analyzed by ELISA (IBL International, 2015). From these three samples, the area under the curve with respect to ground (AUCg) was computed using the trapezoid formula (Pruessner et al., 2003) as an index of total morning cortisol output. The AUCg was the cortisol variable used in all analyses; lower values therefore indicate lower overall morning cortisol secretion. This index reflects total cortisol output across the awakening period and is distinct from the cortisol awakening response (CAR), which captures the dynamic increase over the same interval.

### 2.4. Procedure

The study followed a seven-month longitudinal design with three measurement waves: T1, corresponding to baseline or pre-intervention; T2, corresponding to post-intervention and conducted three months after T1; and T3, corresponding to follow-up and conducted four months after T2.

These intervals were determined according to the duration of the intervention program and the need to conduct the follow-up assessment during a period that did not coincide with school holidays, thereby avoiding distortions in participants’ perceptions of psychosocial working conditions.

During the first phase of the study, educational centers were selected according to educational level and geographical location. Meetings were held at the participating schools to explain the objectives of the research project and request teachers’ voluntary participation.

Before the intervention began, participants attended an informational session during which the structure and objectives of the program were explained. At this stage, participants also received the questionnaires and Salivette collection devices required for cortisol sampling.

For the planned subgroup analyses, baseline questionnaire data collected at T1 were used to classify participants into the four analytical subgroups according to training condition and level of psychosomatic disorders.

### 2.5. Data Analysis

Data analyses were conducted to examine the distributions of the study variables and evaluate changes across the different measurement waves. First, descriptive statistics were calculated for all variables, including means, standard deviations, skewness, and kurtosis, to assess their distributional characteristics. The internal consistency of the scales was also evaluated using Cronbach’s alpha coefficients.

Subsequently, changes in the study variables were examined across the three measurement points: baseline (T1), post-intervention (T2), and follow-up (T3). Paired-samples *t* tests were conducted to compare mean scores across measurement points separately within each analytical group. This approach allowed changes over time to be assessed within each subgroup, defined according to participation in the training program and baseline levels of stress-related health problems.

Specifically, T1 and T2 scores were compared to examine short-term changes following the intervention, whereas T1 and T3 scores were compared to evaluate the persistence of these changes over time. Statistically significant changes in psychosocial demands, resources, burnout, depressive symptoms, and cortisol levels were examined across the different phases of the study. Statistical significance was set at *p* < 0.05. No adjustment for multiple comparisons was applied.

These paired-samples comparisons assess within-group change over time and do not constitute a direct test of whether changes differed significantly between the training and non-training groups. A group × time model (e.g., mixed-effects analysis) was considered but was not conducted because the small analytical subgroup sizes—particularly the focal TG-HHP subgroup (*n* = 13)—would have limited the precision and statistical power of the interaction estimates. Consequently, the analyses should be regarded as exploratory, and the findings should be interpreted as evidence of within-group change rather than as a direct estimate of a between-group intervention effect.

## 3. Results

Internal consistency analyses showed that Cronbach’s alpha coefficients were above 0.70 for all scales at all measurement points (Nunnally, 1978), except for the Indolence dimension, which showed slightly lower reliability at T1 (α = 0.69) and T3 (α = 0.68). Skewness and kurtosis values were within acceptable ranges (±1) for all variables.

### 3.1. Changes Between T1 and T2 (Table 1)

**Figure 1:**
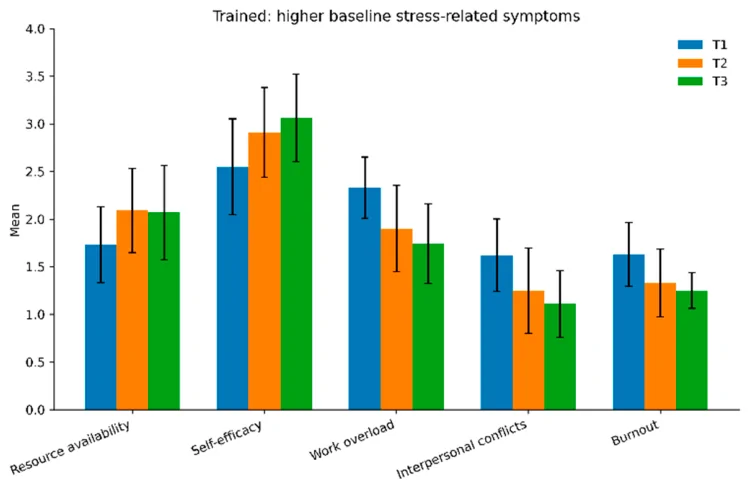
Changes in resources, demands, and burnout over time in the trained group with higher baseline stress-related symptoms. Error bars represent 95% confidence intervals.

**Figure 5:**
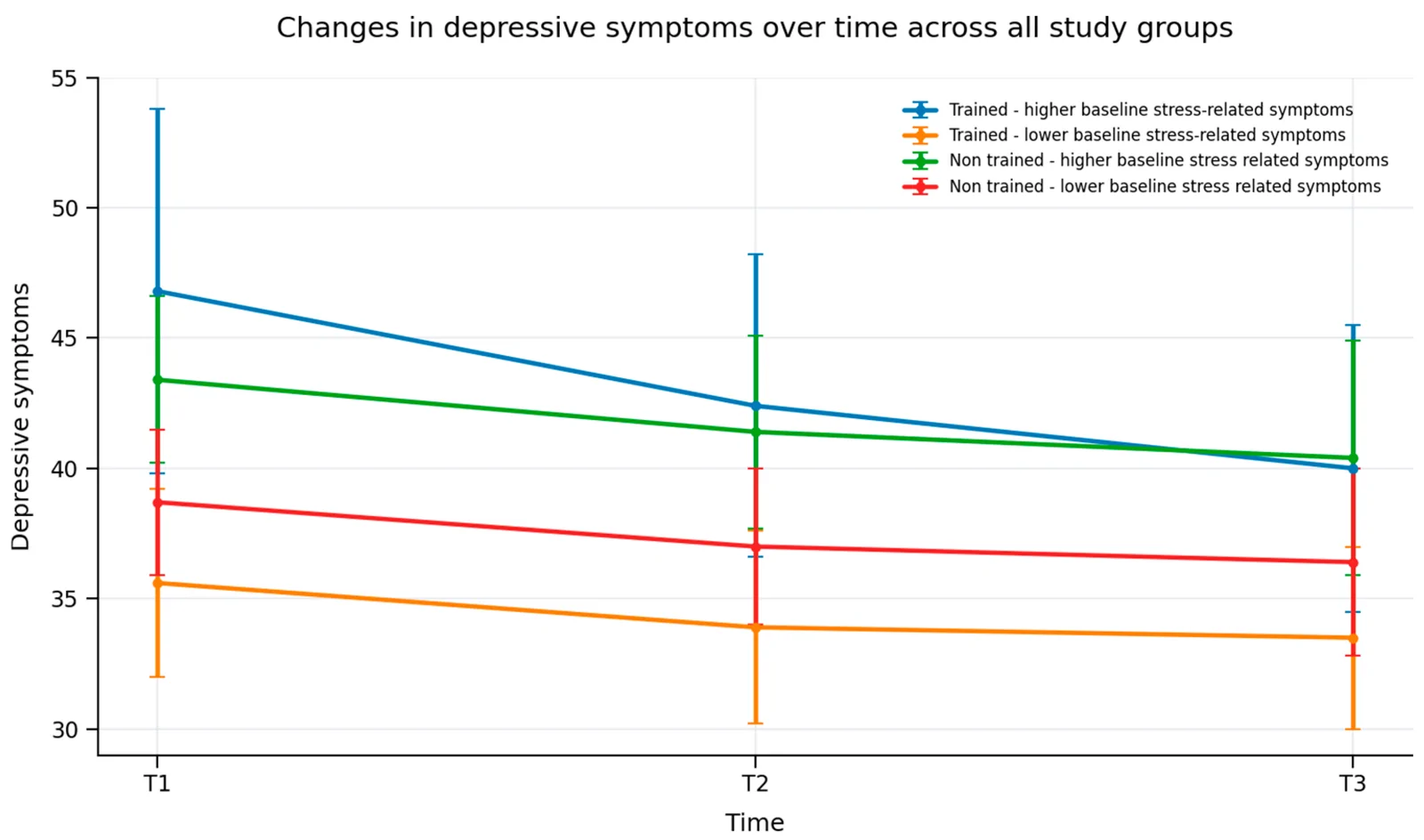
Changes in depressive symptoms over time across all study groups. Error bars represent 95% confidence intervals.

**Figure 6:**
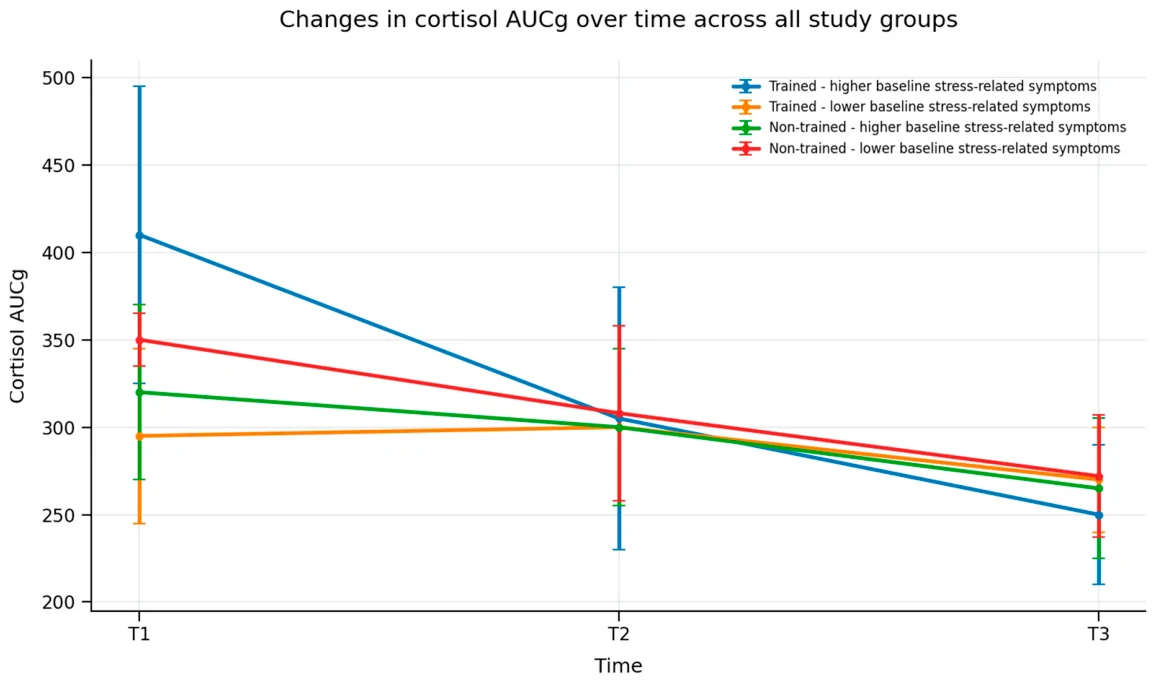
Changes in salivary cortisol AUCg over time across all study groups. Error bars represent 95% confidence intervals.

In the trained group with high baseline stress-related health problems (TG-HHP), statistically significant changes were observed in several variables. Resource availability (t = −3.81, *p* = 0.002, d = −1.06, η^2^ = 0.55) and self-efficacy (t = −3.91, *p* = 0.002, d = −1.09, η^2^ = 0.56) increased significantly, whereas work overload (t = 3.13, *p* = 0.009, d = 0.87, η^2^ = 0.45) and burnout (t = 2.92, *p* = 0.013, d = 0.81, η^2^ = 0.42) decreased significantly. Similarly, depression (t = 2.42, *p* = 0.032, d = 0.67, η^2^ = 0.33) and cortisol (t = 2.82, *p* = 0.018, d = 0.78, η^2^ = 0.40) showed statistically significant reductions. No statistically significant changes were observed in interpersonal conflicts (*p* = 0.089) (Figure 1, Figure 5 and Figure 6).

**Figure 2:**
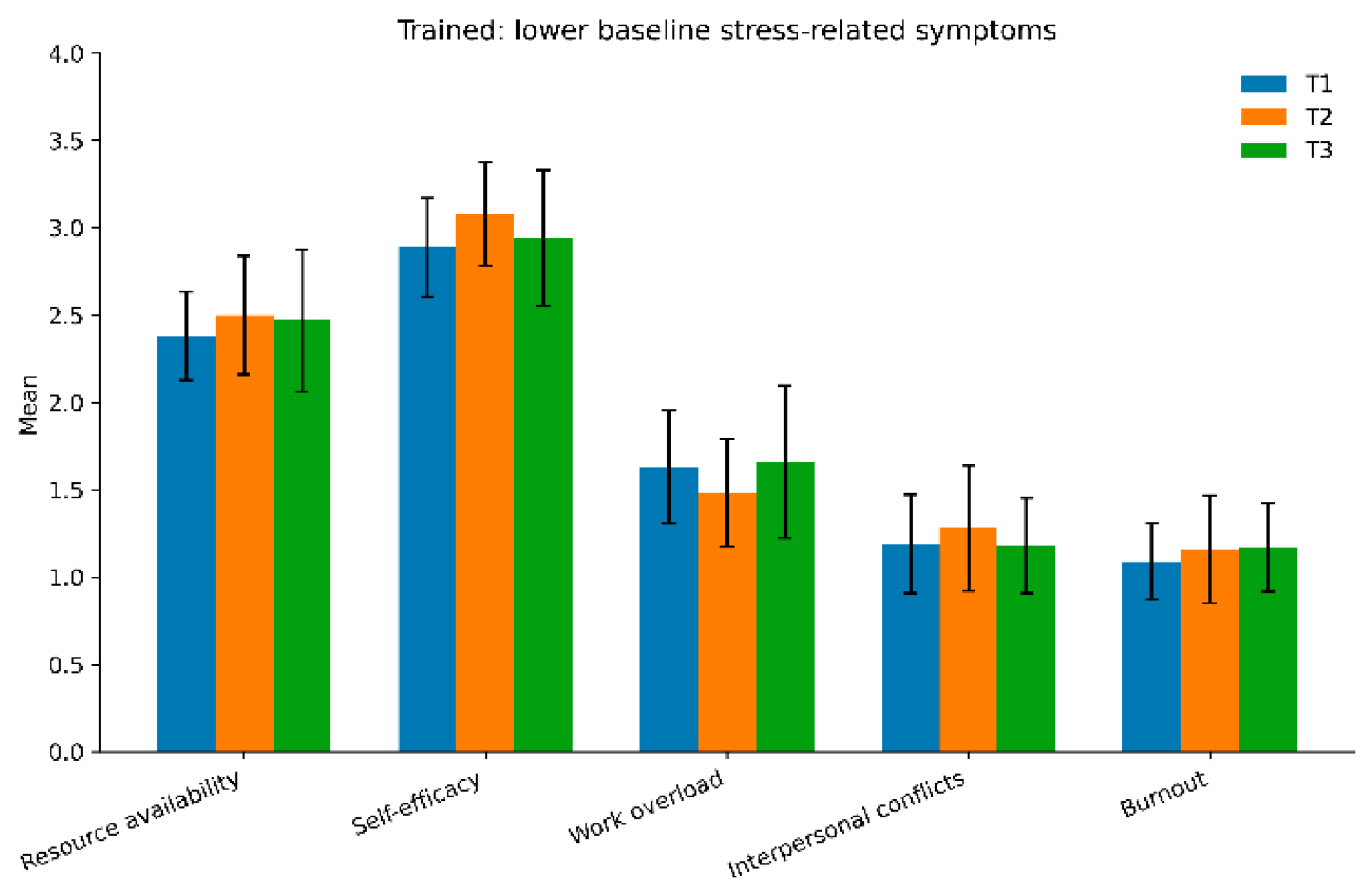
Changes in resources, demands, and burnout over time in the trained group with lower baseline stress-related symptoms. Error bars represent 95% confidence intervals.

In the trained group with low baseline stress-related health problems (TG-LHP), no statistically significant changes were found in any of the variables analyzed (all *p* > 0.05) (Figure 2 and Figure 5).

**Figure 3:**
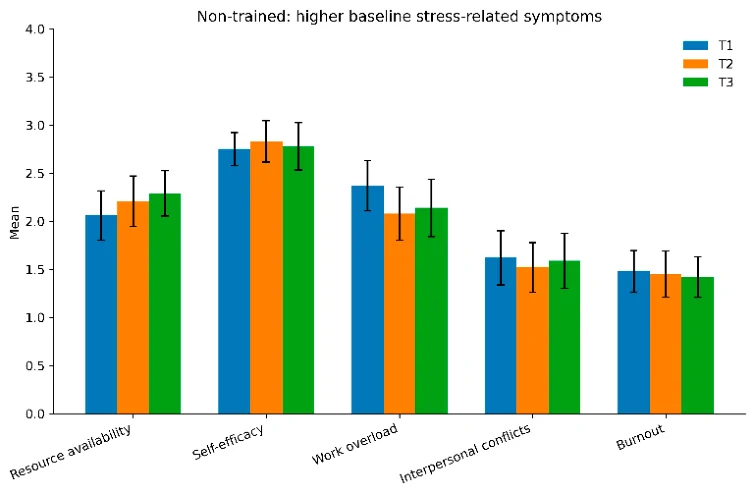
Changes in resources, demands, and burnout over time in the non-trained group with higher baseline stress-related symptoms. Error bars represent 95% confidence intervals.

In the non-trained group with high baseline stress-related health problems (NTG-HHP), a statistically significant reduction was observed in work overload (t = 2.30, *p* = 0.033, d = 0.54, η^2^ = 0.23), whereas no statistically significant changes were found in the remaining variables (Figure 3, Figure 5 and Figure 6).

**Figure 4:**
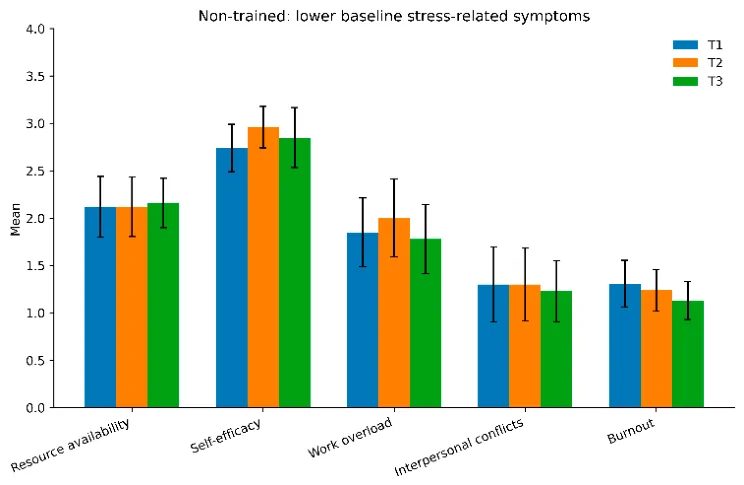
Changes in resources, demands, and burnout over time in the non-trained group with lower baseline stress-related symptoms. Error bars represent 95% confidence intervals.

In the non-trained group with low baseline stress-related health problems (NTG-LHP), a statistically significant increase in self-efficacy was observed (t = −2.73, *p* = 0.016, d = −0.68, η^2^ = 0.32), whereas no statistically significant changes were found in the remaining variables (Figure 4, Figure 5 and Figure 6).

### 3.2. Changes Between T1 and T3 (Table 2)

In the trained group with high baseline stress-related health problems (TG-HHP), statistically significant changes were observed across all variables. Resource availability (t = −2.39, *p* = 0.034, d = −0.66, η^2^ = 0.32) and self-efficacy (t = −4.18, *p* = 0.001, d = −1.16, η^2^ = 0.59) increased significantly. Interpersonal conflicts (t = 2.90, *p* = 0.013, d = 0.81, η^2^ = 0.41) and work overload (t = 3.34, *p* = 0.006, d = 0.93, η^2^ = 0.48) decreased significantly, as did burnout (t = 4.03, *p* = 0.002, d = 1.12, η^2^ = 0.57), depression (t = 2.58, *p* = 0.024, d = 0.71, η^2^ = 0.36), and cortisol (t = 3.09, *p* = 0.010, d = 0.86, η^2^ = 0.44).

In the trained group with low baseline stress-related health problems (TG-LHP), no statistically significant changes were observed in any of the variables analyzed (all *p* > 0.05).

In the non-trained group with high baseline stress-related health problems (NTG-HHP), no statistically significant changes were found between T1 and T3 (all *p* > 0.05).

In the non-trained group with low baseline stress-related health problems (NTG-LHP), no statistically significant changes were observed in any of the variables.

Overall, the largest number of statistically significant within-group changes was observed in the TG-HHP group.

## 4. Discussion

This longitudinal study examined changes associated with participation in a multicomponent cognitive–behavioral intervention aimed at reducing psychosocial risks, burnout, depressive symptoms, and physiological stress among teachers. The clearest pattern of findings was observed among trained teachers who reported high levels of stress-related health problems at baseline. In this subgroup, participation in the program was accompanied by increases in perceived resource availability and self-efficacy; reductions in workload, interpersonal conflicts, burnout, and depressive symptoms; and lower salivary cortisol levels. Most of these changes were maintained at the four-month follow-up. By contrast, no comparable and consistent pattern of change emerged among trained teachers with low baseline health problems or among the two non-trained groups. Taken together, these findings provide preliminary evidence that the potential benefits of teacher-focused stress interventions may depend partly on participants’ initial levels of vulnerability and may extend across psychosocial, psychological, and physiological domains (Agyapong et al., 2023; Gaab et al., 2003).

The concentration of changes in the trained group with high baseline stress-related health problems (TG-HHP) is consistent with the theoretical rationale underlying the study. Within the Job Demands–Resources model, strain develops when sustained job demands exceed the personal and organizational resources available to manage them (Bakker & Demerouti, 2017; Demerouti et al., 2001). Teachers experiencing greater stress-related health problems may therefore have had both a stronger need for intervention and greater scope for measurable improvement. This interpretation is also consistent with previous research suggesting that the effects of stress-management interventions may be more apparent among individuals with elevated baseline distress or vulnerability (Gaab et al., 2006; Manigault et al., 2019). From this perspective, the increase in perceived resources and self-efficacy may reflect an improvement in participants’ capacity to recognize, mobilize, and use the resources available within their work environment (Ansley et al., 2021; Täschner et al., 2024).

The intervention was not designed to modify the organizational conditions of the participating schools. Rather, it targeted how teachers evaluated and managed their professional demands. Its components—including cognitive restructuring, problem-solving, emotional regulation, role negotiation, self-organization, and social support (Gil-Monte, 2019b)—were designed to modify both the appraisal of stressful situations and the strategies used to respond to them. The reductions in perceived workload and interpersonal conflicts are therefore consistent with an appraisal- and coping-based mechanism, even in the absence of documented changes in objective working conditions (Forman, 1982; Żołnierczyk-Zreda, 2005). Nevertheless, the present study did not include specific process measures capable of establishing whether changes in cognitive appraisal, irrational beliefs, coping behavior, or role negotiation mediated the observed outcomes. These mechanisms should consequently be regarded as theoretically plausible interpretations rather than empirically demonstrated pathways.

The magnitude and consistency of the within-group changes in the TG-HHP are also noteworthy. Effect-size estimates were substantial across several conceptually related outcomes, particularly self-efficacy, burnout, workload, and cortisol. These estimates must be interpreted cautiously because they were derived from a small subgroup and may therefore be unstable. Even so, the fact that changes occurred in the expected direction across resources, demands, psychological outcomes, and a physiological indicator suggests a coherent pattern rather than an isolated finding restricted to a single measure. This pattern is broadly consistent with previous evidence indicating that cognitive–behavioral and multicomponent programs can reduce occupational stress and burnout among teachers and other helping professionals (Agyapong et al., 2023; Denuwara et al., 2022; Maricuţoiu et al., 2016; Paudel et al., 2022; Richardson & Rothstein, 2008). Direct comparisons between effect sizes should, however, be avoided because of differences in sample characteristics, intervention content, study design, and analytical procedures.

The maintenance of most changes at T3, four months after completion of the program, suggests that the observed improvements were not limited to an immediate post-intervention response. Resource availability and self-efficacy remained higher than at baseline, while perceived demands, burnout, depressive symptoms, and cortisol remained lower. This temporal pattern is compatible with the possibility that participants continued to apply some of the strategies acquired during the program in their daily professional activities (Forman, 1990; Hammerfald et al., 2006). At the same time, the findings do not demonstrate enduring cognitive restructuring or long-term behavioral change, as the study did not directly assess the use of intervention techniques after the program ended. The follow-up therefore supports the medium-term stability of the measured outcomes, although longer assessment periods would be required to establish whether these changes persist over time.

The concurrent reductions in burnout and depressive symptoms in the TG-HHP are particularly relevant. Although burnout and depression are related, they are conceptually and empirically distinguishable constructs (Koutsimani et al., 2019; Parker & Tavella, 2021; Tavella et al., 2023). Burnout is specifically linked to chronic occupational stress, whereas depressive symptoms extend beyond the work context. Their joint reduction is nevertheless consistent with evidence that both may arise partly from overlapping processes, including persistent stress exposure, psychological exhaustion, negative self-evaluation, and maladaptive coping (Ghasemi et al., 2024; Maslach & Leiter, 2016; Rothe et al., 2020). The intervention may therefore have influenced processes that contribute to both occupational burnout and broader emotional distress. However, because both variables were assessed through self-report, part of their parallel evolution may also reflect shared measurement variance or a more general improvement in perceived well-being.

One of the distinctive features of the study is the incorporation of salivary cortisol alongside self-reported psychosocial and psychological outcomes. The reduction in cortisol observed in the TG-HHP at post-intervention and follow-up is consistent with previous research suggesting that psychological interventions may be accompanied by changes in physiological stress regulation (Gaab et al., 2003; Hammerfald et al., 2006; Romero-González et al., 2020). The convergence between lower cortisol levels and improvements in burnout, depression, demands, and resources provides a broader picture of the changes associated with participation in the program than would have been obtained through self-report alone.

This physiological result should nevertheless be interpreted prudently. Salivary cortisol is influenced by sleep, awakening time, food and caffeine intake, physical activity, menstrual factors, medication, and adherence to the sampling procedure (Chida & Steptoe, 2009; Fries et al., 2009; Karlson et al., 2011). Although participants were instructed to collect samples immediately upon awakening and 15 and 30 min thereafter (Fries et al., 2009), adherence to the prescribed sampling times could not be independently verified (Karlson et al., 2011). In addition, downward trends were also observed among some participants who did not receive the intervention, suggesting that temporal or contextual factors may have contributed to part of the change. Accordingly, the cortisol findings should be understood as convergent physiological evidence compatible with reduced stress activation rather than as definitive proof that the intervention directly modified HPA-axis functioning.

The absence of a consistent pattern of statistically significant changes in the remaining groups also requires a balanced interpretation. Among trained teachers with low baseline health problems, the lack of substantial change may reflect a floor effect or limited room for improvement. Intensive stress-management programs may generate fewer detectable benefits when participants begin with relatively favorable levels of health and psychosocial functioning. This supports the possibility that needs-based or stratified interventions may be more efficient than applying the same intensive program uniformly to all teachers (Estevez Cores et al., 2021; Gaab et al., 2006; Manigault et al., 2019).

At the same time, the absence of statistically significant changes cannot, by itself, establish that these groups were unaffected. Their small sizes limited the statistical power to detect modest effects. Moreover, isolated changes in workload among non-trained teachers with high baseline stress-related health problems and in self-efficacy among non-trained teachers with low baseline stress-related health problems indicate that the comparison conditions were not entirely stable. These findings reinforce the need to distinguish between the absence of statistical significance and evidence of no effect. They also caution against attributing every change observed in the TG-HHP exclusively to the intervention.

From the perspective of the Job Demands–Resources model, the findings are consistent with the proposition that greater access to personal and occupational resources, together with a more manageable perception of job demands, is associated with lower psychological strain (Bakker & Demerouti, 2017; Demerouti et al., 2001). The present study does not, however, constitute a formal test of the structural relationships proposed by the model. The JD–R framework was used to select the psychosocial variables targeted by the intervention and to interpret their joint evolution. Future research should examine whether increases in self-efficacy or perceived resources mediate reductions in burnout and depressive symptoms and whether baseline strain moderates the magnitude of intervention-related change.

The study also has implications for teacher professional development and educational psychology. Programs intended to improve educational quality have traditionally focused primarily on pedagogical competence, instructional methods, and classroom management (Jennings & DeMauro, 2017; Kyriacou, 2001). However, teachers’ capacity to regulate occupational stress and maintain their psychological health may also influence their professional functioning, relationships with students and colleagues, and engagement with teaching (Madigan et al., 2023; Skaalvik & Skaalvik, 2021). In this respect, interventions addressing teachers’ psychosocial resources and coping processes may complement more conventional forms of professional development. From an applied behavioral perspective, integrating stress-management competencies into initial and in-service teacher training—particularly for teachers identified as being at higher risk—could complement the pedagogical and instructional focus that has traditionally dominated professional development programs.

The results suggest that the allocation of intensive preventive resources according to baseline vulnerability deserves further examination. In the present sample, the clearest changes occurred among teachers who initially reported more stress-related health problems, whereas teachers with lower baseline vulnerability showed little measurable change. This does not imply that universal preventive initiatives lack value, but it suggests that their intensity and content may need to be adapted to teachers’ initial needs. This pattern should be understood as a hypothesis to be confirmed in larger randomized studies rather than as evidence that teachers at lower baseline risk do not benefit from such programs; the absence of statistically significant change in lower-vulnerability subgroups may partly reflect limited statistical power and reduced scope for improvement. Screening procedures followed by targeted intervention could potentially improve the efficiency of occupational health programs in educational settings (Estevez Cores et al., 2021; Gaab et al., 2006).

The multicomponent nature of the program may also be relevant to its educational applicability. Teacher stress is rarely attributable to a single process; it often involves emotional demands, maladaptive cognitions, organizational difficulties, interpersonal conflicts, insufficient recovery, and limited perceived support (Kyriacou, 2001; Madigan et al., 2023; Skaalvik & Skaalvik, 2021). Addressing these dimensions within an integrated program may therefore be more appropriate than relying on a single technique (Wiederhold et al., 2018). Recent work in Mexican university settings has implemented our program with positive outcomes, adopting its multicomponent approach that combines psychological, organizational, and physiological strategies to address burnout among teaching staff (Unda Rojas et al., 2026).

The present design does not allow the specific contribution of each component to be isolated. The evaluation therefore addresses the program as an integrated unit, consistent with how it is intended to be delivered in practice, but leaves the question of active ingredients open for future dismantling studies.

### Limitations

The interpretation of these findings should be tempered by several limitations.

First, the study used a quasi-experimental design without random assignment. Participation in the training program was voluntary, and self-selection may have produced differences between trained and non-trained participants that were not fully captured by the baseline comparisons. Although the groups were examined in relation to relevant sociodemographic, occupational, behavioral, medication-related, and health variables, residual confounding cannot be excluded. Consequently, the findings support an association between participation in the program and the observed changes, but they do not permit definitive causal attribution.

Second, the overall sample was modest, and the analytical subgroups contained between 13 and 20 participants. These small cell sizes reduced statistical power, particularly in the groups in which no statistically significant changes were found, and may have produced unstable effect-size estimates. The use of convenience sampling and the predominance of women in the sample also limit the extent to which the findings can be generalized to other teacher populations, educational systems, or occupational contexts.

Third, the analytical strategy relied primarily on paired-samples comparisons within each subgroup. This approach allowed changes between baseline, post-intervention, and follow-up to be examined in relation to the study hypotheses, but it did not directly test the interaction between intervention condition, baseline vulnerability, and time. In addition, the number of comparisons increased the possibility of Type I error. The theoretically motivated classification according to baseline health problems also reduced a continuous variable to categorical groups. Moreover, because the focal subgroup was defined by elevated baseline levels of psychosomatic disorders, regression to the mean cannot be excluded as a partial explanation for the magnitude of the changes observed in this subgroup. Future studies with larger samples should employ mixed-effects or comparable longitudinal models, directly test group × time interactions, and examine baseline psychosomatic symptom scores as a continuous moderator.

Fourth, most outcomes were assessed through self-report, making the results potentially susceptible to common-method variance and response expectations. The inclusion of salivary cortisol strengthened the design by providing a physiological indicator, but cortisol is itself sensitive to contextual and behavioral influences that could not be completely controlled under field conditions. Future studies would benefit from combining self-reports and biomarkers with objective indicators of working conditions, behavioral outcomes, and more detailed monitoring of cortisol sampling.

These limitations do not eliminate the relevance of the observed pattern, but they define the level at which it should be interpreted. The study provides preliminary evidence that a structured cognitive–behavioral program may be associated with multidimensional improvements among teachers with elevated stress-related health problems. Replication in larger randomized studies using analytical models designed to compare trajectories directly is necessary before firm conclusions regarding efficacy, mechanisms, or generalizability can be drawn.

## 5. Conclusions

The findings of this longitudinal quasi-experimental study suggest that teachers with elevated baseline levels of stress-related health problems may derive particular benefit from a structured multicomponent cognitive–behavioral intervention. Within this subgroup, participation in the program was associated with increases in perceived resource availability and self-efficacy; reductions in perceived workload and interpersonal conflicts; and decreases in burnout, depressive symptoms. Morning cortisol AUCg also decreased in this subgroup; given its sensitivity to contextual and behavioral factors, this physiological change should be interpreted as convergent evidence compatible with reduced stress activation rather than as definitive proof of a direct intervention effect on HPA-axis functioning. Most of these changes were maintained at the four-month follow-up.

No similarly consistent pattern was observed among trained teachers with lower baseline stress-related health problems or among the non-trained comparison groups. This distribution of findings supports further investigation of stratified, needs-based approaches to teacher stress prevention, in which the intensity of the intervention is adapted to participants’ initial vulnerability.

Given the non-randomized design, modest subgroup sizes, and analytical limitations, the results should be regarded as preliminary rather than conclusive evidence of intervention efficacy. Nevertheless, the convergence of psychosocial, psychological, and physiological changes highlights the value of evaluating occupational stress interventions through multiple indicators. The study therefore contributes to the literature on teacher well-being by identifying a potentially relevant target group and providing a basis for larger randomized studies of targeted intervention programs in educational settings.

## Figures and Tables

**Table 1:** Paired-samples comparisons between T1 and T2 across the four study groups.

Group	Variable	T1 M (SD)	T2 M (SD)	t	*p*	d	η^2^
TG-HHP	Resource availability	1.73 (0.66)	2.09 (0.73)	−3.81	0.002 *	−1.06	0.55
TG-HHP	Self-efficacy	2.55 (0.83)	2.91 (0.78)	−3.91	0.002 *	−1.09	0.56
TG-HHP	Interpersonal conflicts	1.62 (0.63)	1.25 (0.74)	1.85	0.089	0.51	0.22
TG-HHP	Work overload	2.33 (0.53)	1.90 (0.75)	3.13	0.009 *	0.87	0.45
TG-HHP	Burnout	1.63 (0.55)	1.33 (0.59)	2.92	0.013 *	0.81	0.42
TG-HHP	Depression	46.92 (11.66)	42.40 (9.64)	2.42	0.032 *	0.67	0.33
TG-HHP	Cortisol	410.01 (146.09)	306.50 (119.26)	2.82	0.018 *	0.78	0.40
TG-LHP	Resource availability	2.38 (0.49)	2.50 (0.66)	−1.11	0.282	−0.29	0.08
TG-LHP	Self-efficacy	2.89 (0.55)	3.08 (0.58)	−1.96	0.068	−0.51	0.20
TG-LHP	Interpersonal conflicts	1.19 (0.55)	1.28 (0.70)	−0.61	0.553	−0.16	0.03
TG-LHP	Work overload	1.63 (0.63)	1.48 (0.60)	1.30	0.212	0.34	0.10
TG-LHP	Burnout	1.09 (0.43)	1.16 (0.60)	−0.82	0.427	−0.21	0.05
TG-LHP	Depression	35.69 (7.05)	33.88 (7.03)	1.57	0.136	0.41	0.14
TG-LHP	Cortisol	296.87 (95.87)	304.85 (93.90)	−0.23	0.824	−0.06	0.00
NTG-HHP	Resource availability	2.06 (0.55)	2.21 (0.56)	−1.27	0.220	−0.30	0.09
NTG-HHP	Self-efficacy	2.75 (0.37)	2.83 (0.46)	−0.89	0.383	−0.21	0.05
NTG-HHP	Interpersonal conflicts	1.62 (0.60)	1.52 (0.55)	0.79	0.440	0.19	0.03
NTG-HHP	Work overload	2.37 (0.56)	2.08 (0.59)	2.30	0.033 *	0.54	0.23
NTG-HHP	Burnout	1.48 (0.46)	1.45 (0.51)	0.44	0.665	0.10	0.01
NTG-HHP	Depression	43.44 (6.67)	41.42 (8.04)	1.64	0.118	0.38	0.14
NTG-HHP	Cortisol	318.31 (101.30)	300.25 (97.86)	0.57	0.577	0.13	0.02
NTG-LHP	Resource availability	2.12 (0.60)	2.12 (0.59)	−0.05	0.963	-0.01	0.00
NTG-LHP	Self-efficacy	2.74 (0.47)	2.96 (0.41)	−2.73	0.016 *	-0.68	0.32
NTG-LHP	Interpersonal conflicts	1.30 (0.74)	1.30 (0.72)	0.00	1.000	0.00	0.00
NTG-LHP	Work overload	1.85 (0.68)	2.00 (0.77)	−0.87	0.399	−0.22	0.05
NTG-LHP	Burnout	1.31 (0.46)	1.24 (0.41)	1.10	0.291	0.27	0.07
NTG-LHP	Depression	38.63 (5.29)	37.03 (5.49)	1.71	0.108	0.43	0.15
NTG-LHP	Cortisol	350.51 (113.06)	310.18 (96.56)	0.98	0.349	0.24	0.07

Note 1. TG-LHP = trained group with low baseline stress-related health problems; TG-HHP = trained group with high baseline stress- related health problems; NTG-LHP = non-trained group with low baseline stress-related health problems; NTG-HHP = non-trained group with high baseline stress-related health problems. Paired-samples *t* tests were used. Note 2. * *p* < 0.05. d = Cohen’s d; η^2^ = eta squared.

**Table 2:** Paired-samples comparisons between T1 and T3 across the four study groups.

Group	Variable	T1 M (SD)	T3 M (SD)	t	*p*	d	η^2^
TG-HHP	Resource availability	1.73 (0.66)	2.07 (0.82)	−2.39	0.034 *	−0.66	0.32
TG-HHP	Self-efficacy	2.55 (0.83)	3.06 (0.76)	−4.18	0.001 *	−1.16	0.59
TG-HHP	Interpersonal conflicts	1.62 (0.63)	1.11 (0.58)	2.90	0.013 *	0.81	0.41
TG-HHP	Work overload	2.33 (0.53)	1.74 (0.69)	3.34	0.006 *	0.93	0.48
TG-HHP	Burnout	1.63 (0.55)	1.25 (0.31)	4.03	0.002 *	1.12	0.57
TG-HHP	Depression	46.92 (11.66)	40.00 (9.18)	2.58	0.024 *	0.71	0.36
TG-HHP	Cortisol	389.01 (157.15)	250.20 (67.38)	3.09	0.010 *	0.86	0.44
TG-LHP	Resource availability	2.38 (0.49)	2.47 (0.79)	−0.60	0.555	−0.15	0.03
TG-LHP	Self-efficacy	2.89 (0.55)	2.94 (0.76)	−0.43	0.670	−0.11	0.01
TG-LHP	Interpersonal conflicts	1.19 (0.55)	1.18 (0.53)	0.09	0.929	0.02	0.00
TG-LHP	Work overload	1.63 (0.63)	1.66 (0.85)	−0.23	0.818	−0.06	0.00
TG-LHP	Burnout	1.09 (0.43)	1.17 (0.49)	−1.09	0.290	−0.28	0.08
TG-LHP	Depression	35.69 (7.05)	33.47 (6.76)	1.84	0.085	0.47	0.19
TG-LHP	Cortisol	312.41 (92.39)	272.34 (54.24)	1.44	0.174	0.37	0.13
NTG-HHP	Resource availability	2.08 (0.58)	2.29 (0.49)	−1.74	0.100	−0.41	0.15
NTG-HHP	Self-efficacy	2.71 (0.37)	2.78 (0.51)	−0.58	0.567	−0.14	0.02
NTG-HHP	Interpersonal conflicts	1.60 (0.61)	1.59 (0.59)	0.08	0.941	0.02	0.00
NTG-HHP	Work overload	2.37 (0.58)	2.14 (0.62)	1.49	0.154	0.35	0.10
NTG-HHP	Burnout	1.46 (0.46)	1.42 (0.44)	0.42	0.679	0.10	0.01
NTG-HHP	Depression	43.37 (6.33)	40.44 (9.26)	1.74	0.100	0.41	0.15
NTG-HHP	Cortisol	311.50 (107.14)	263.03 (57.16)	1.60	0.131	0.38	0.13
NTG-LHP	Resource availability	2.22 (0.45)	2.16 (0.47)	0.46	0.653	0.11	0.01
NTG-LHP	Self-efficacy	2.77 (0.47)	2.85 (0.57)	−0.57	0.575	−0.14	0.02
NTG-LHP	Interpersonal conflicts	1.25 (0.74)	1.23 (0.58)	0.17	0.865	0.04	0.00
NTG-LHP	Work overload	1.83 (0.70)	1.78 (0.66)	0.57	0.576	0.14	0.02
NTG-LHP	Burnout	1.32 (0.48)	1.13 (0.36)	2.08	0.056	0.51	0.23
NTG-LHP	Depression	38.27 (5.27)	36.47 (6.52)	2.13	0.052	0.52	0.24
NTG-LHP	Cortisol	343.42 (116.74)	270.96 (64.58)	2.10	0.059	0.51	0.23

Note 1. TG-LHP = trained group with low baseline stress-related health problems; TG-HHP = trained group with high baseline stress-related health problems; NTG-LHP = non-trained group with low baseline stress-related health problems; NTG-HHP = non-trained group with high baseline stress-related health problems. Paired-samples *t* tests were used. Note 2. * *p* < 0.05. d = Cohen’s d; η^2^ = eta squared.

## Data Availability

The raw data supporting the conclusions of this article will be made available by the authors upon request.
